# Effect of lung transplantation on biventricular function in pulmonary arterial hypertension: A systematic review

**DOI:** 10.1016/j.jhlto.2026.100715

**Published:** 2026-10-27

**Authors:** Adil Sarvar Mohammed, Priyatham Gurram, Sai Priyanka Mellacheruvu, Sai Prasanna Lekkala, Maya Asami Takagi, Sai Gautham Kanagala, Umera Yasmeen, Hamdi Lababidi, Zainab J. Gandhi, Bilal Khan Mohammed, Shweta Kambali, Rupak Desai, Shrinivas Kambali

**Affiliations:** a Department of Internal Medicine, Central Michigan University College of Medicine, Saginaw, MI; b Department of Internal Medicine, Trinity Health – Nazareth Hospital, Philadelphia, PA; c Department of Internal Medicine, UCHealth Parkview Medical Center, Pueblo, CO; d Department of Internal Medicine, University of California Davis Medical Center, Sacramento, CA; e Department of Internal Medicine, New York Medical College / Metropolitan Hospital Center, New York, NY; f Mamata Medical College, D.No.5, 7-200/8/A, Mamatha Hospital Rd, Manikya Nagar Basti, Khammam, Telangana, India; g Department of Pulmonary and Critical Care, Cleveland Clinic Foundation, Cleveland, OH; h Department of Cardiac Surgery, Northwestern University Feinberg School of Medicine, Chicago, IL; i Department of Internal Medicine, McLaren Bay Region, Bay City, MI; j Independent Outcomes Researcher, Atlanta, GA; k Department of Pulmonary and Critical Care, MyMichigan Medical Center, Saginaw, MI

**Keywords:** pulmonary arterial hypertension, lung transplantation, right ventricular function, left ventricular dysfunction, cardiac MRI, echocardiography, extracorporeal membrane oxygenation

## Abstract

Pulmonary arterial hypertension (PAH) leads to progressive right ventricular (RV) failure and secondary left ventricular (LV) dysfunction due to chronic pressure overload and ventricular interdependence. Lung transplantation (LTx) is an established treatment for end-stage PAH, yet patterns of biventricular recovery and the role of veno-arterial perioperative extracorporeal membrane oxygenation (VA-ECMO) remain incompletely defined. A PubMed search identified studies published from 1990 to 2025 using MeSH terms related to lung transplantation, ventricular function, echocardiography, cardiac MRI, and ECMO. Prospective studies, retrospective analyses, and case series evaluating post-transplant cardiac function in PAH were included. A total of 13 studies were included. Across studies, LTx resulted in rapid and substantial RV reverse remodeling, with improvements in RV size, mass, systolic function, tricuspid regurgitation, and pulmonary pressures. This reduction in RV afterload was accompanied by progressive improvement of RV output. In contrast, LV recovery was more variable, possibly due to chronic underfilling and LV remodeling. Cardiac MRI and echocardiography demonstrated progressive biventricular structural and functional adaptation over time. Perioperative VA-ECMO may provide hemodynamic support during this transition by unloading the RV and maintaining perfusion while ventricular loading conditions rapidly change. Lung transplantation for PAH initiates transition from chronic RV pressure overload and LV underfilling to improved pulmonary vascular conditions and subsequent biventricular recovery. Further longitudinal studies are needed to identify predictors of maladaptive LV responses and determine which patients may benefit from prolonged perioperative VA-ECMO support.

## Highlights

• RV reverse remodeling after lung transplantation for PAH is early and consistent
• Left ventricular responses vary; early dysfunction is common but usually transient
• Disagreement between studies reflects imaging timing and dysfunction definitions
• Chronic LV underfilling explains poor tolerance of abruptly restored preload
• No controlled study has tested whether prolonged VA-ECMO improves outcomes

## Introduction

Pulmonary arterial hypertension (PAH) is a progressive disease characterized by elevated pulmonary vascular resistance, with an estimated annual incidence of 5.8 cases per million population.^1^ Hemodynamically, pre-capillary pulmonary hypertension (PH) is defined by a mean pulmonary arterial pressure >20 mm Hg at rest, a pulmonary vascular resistance (PVR) >2 Wood units, and a pulmonary arterial wedge pressure ≤15 mm Hg.^2^ PAH (Group 1 PH) is a clinical diagnosis made within this hemodynamic definition: it additionally requires classification according to the clinical groups of PH and exclusion of the other causes of pre-capillary PH, namely PH associated with lung disease or hypoxia (Group 3), chronic thromboembolic PH (Group 4), and PH with unclear or multifactorial mechanisms (Group 5).^2^ Lung transplantation is an established option for advanced Group 1 PAH that progresses despite maximal medical therapy, and this cohort is the focus of the present review.^3^, ^4^ The place of transplantation differs across the other clinical groups. Group 2 PH is managed by treating the underlying left heart disease, and Group 4 disease by pulmonary endarterectomy, balloon pulmonary angioplasty, or targeted medical therapy.^2^ In Group 3, by contrast, severe PH complicating advanced chronic lung disease is itself an indication for lung transplant referral and listing in eligible patients, and it is the combination of parenchymal disease and PH, rather than either alone, that defines candidacy.^4^

Advanced PAH results in substantial cardiac remodeling, particularly of the right ventricle (RV), including RV dilation, hypertrophy, and tricuspid regurgitation. Chronic RV pressure overload also affects the left ventricle (LV) through ventricular interdependence, leading to LV dysfunction (LVD).^5^, ^6^ Lung transplantation (LTx) is an established therapeutic option for end-stage PAH. Bilateral lung transplantation (BLT) is preferred over heart–lung transplantation (HLT) due to comparable outcomes and conservation of donor organs, although HLT remains necessary for select patients with complex congenital heart disease or severely enlarged native hearts that preclude implantation of donor lungs.^4^

Several factors have been associated with poor outcomes in PAH patients undergoing LTx, including syncope, hyponatremia, low cardiac index, and an enlarged right-to-left ventricular index.^7^ Evidence consistently demonstrates substantial improvement in RV function after transplantation; however, recovery of LV function is less well understood. Post-operative complications are more common among PAH recipients, with primary graft dysfunction (PGD) from RV back pressure, pulmonary edema, bleeding, infection, and LVD remaining the leading causes of morbidity and mortality.^8^, ^9^, ^10^, ^11^ Post-operative transient LVD—characterized by reduced LV ejection fraction (LVEF), impaired diastolic function, and decreased LV free-wall mass—may result from the sudden increase in preload to a previously underfilled LV after normalization of PVR.^12^

Existing syntheses address parts of this problem separately. Consensus documents on candidate selection establish when transplantation should be offered and to whom,^3^, ^4^ and recent International Society for Heart and Lung Transplantation (ISHLT) consensus statements set out how extracorporeal life support (ECLS) should be configured, monitored, and weaned across the preoperative, intraoperative, and postoperative phases. Narrative reviews of the transplanted PAH recipient concentrate on RV recovery and on prognosis. What none of them provides is an account of the LV across the same period or an explicit link between the timing of the preload transition and the extracorporeal strategy used to cross it. We identified no prior systematic review of biventricular imaging outcomes in this population.

Given the limited data characterizing LV recovery after LTx in PAH, this review focuses on the integrated process of biventricular recovery following abrupt normalization of PVR. We synthesize evidence describing the transition from chronic RV pressure overload and LV underfilling to rapid RV reverse remodeling, increased LV preload, and subsequent LV adaptation after transplantation. We also examine how echocardiography and cardiac magnetic resonance (CMR) characterize these changes over time, and how extracorporeal membrane oxygenation (ECMO), and in particular veno-arterial ECMO (VA-ECMO), may modulate this hemodynamic transition in patients at risk for early ventricular dysfunction.

## Methods

### Information sources and search strategy

A comprehensive literature search was conducted in PubMed/MEDLINE covering the period from January 1st, 1990, to May 31st, 2025. Search terms incorporated Medical Subject Headings (MeSH) along with free-text keywords covering three main thematic domains: PH, LTx, and cardiac metrics encompassing ventricular structure, mechanical function, hemodynamics, noninvasive imaging, and mechanical circulatory support via ECMO, joined using the logical operator AND. Search parameters were executed without restrictions regarding publication language or document format. Comprehensive details of the retrieval protocol are outlined in Supplementary Table S1. Furthermore, manual reference screening of selected investigations and pertinent consensus statements was conducted alongside forward citation tracking.

### Eligibility criteria and outcomes

We included prospective and retrospective cohort studies, case–control studies, and case series of two or more adults undergoing LTx for PAH, including primary or idiopathic PH and Eisenmenger syndrome, that reported right or left ventricular structure, function, remodeling, or invasive hemodynamics after transplantation, or perioperative ECMO in relation to ventricular outcomes. The minimum series size was set at two patients because transplantation for PAH is uncommon, and several of the earliest descriptions of early postoperative LVD appeared in very small series; in the event, no included study enrolled fewer than 10 patients, so this threshold did not affect the final evidence base. Cohorts of mixed PH etiology were eligible where patients with PH formed a defined part of the transplanted cohort and ventricular outcomes were reported for that cohort; where a PAH subgroup could not be separated, the study is identified as contributing indirect evidence. We excluded pediatric cohorts, animal studies, transplant candidates without post-transplant assessment, isolated HLT, single case reports, and publications not available in English. Full criteria are given in Supplementary Table S2.

The primary endpoints evaluated were post-transplant alterations in RV geometry and function (comprising ejection fraction, chamber dimensions, mass, fractional area change, Tricuspid annular plane systolic excursion (TAPSE), pulmonary artery systolic pressures (PASP), tricuspid regurgitation severity, and right ventricle-pulmonary artery (RV-PA) coupling) alongside LV structural and functional dynamics (including ejection fraction, volume, mass index, diastolic filling parameters, cardiac index, and transient postoperative LV failure). Secondary endpoints encompassed the temporal kinetics of biventricular recovery, interventricular septal mechanics, and the influence of perioperative VA-ECMO on ventricular adaptation, hemodynamic stability, PGD, and early post-transplant survival.

### Study selection and characteristics

The search returned 998 records, none of which were duplicates. After title and abstract screening, 920 were excluded, and 78 full-text reports were assessed, of which 65 were excluded: 31 reported no post-transplant ventricular or hemodynamic outcome, 19 reported no ventricular or hemodynamic data attributable to patients with PH, 8 did not report lung transplant recipients or post-transplant assessment, 6 were case reports or provided insufficient data, and 1 studied a pediatric population. A further 5 reports identified by citation searching were assessed, and none met the eligibility criteria. Thirteen studies were included (Figure 1).

**Figure 1:**
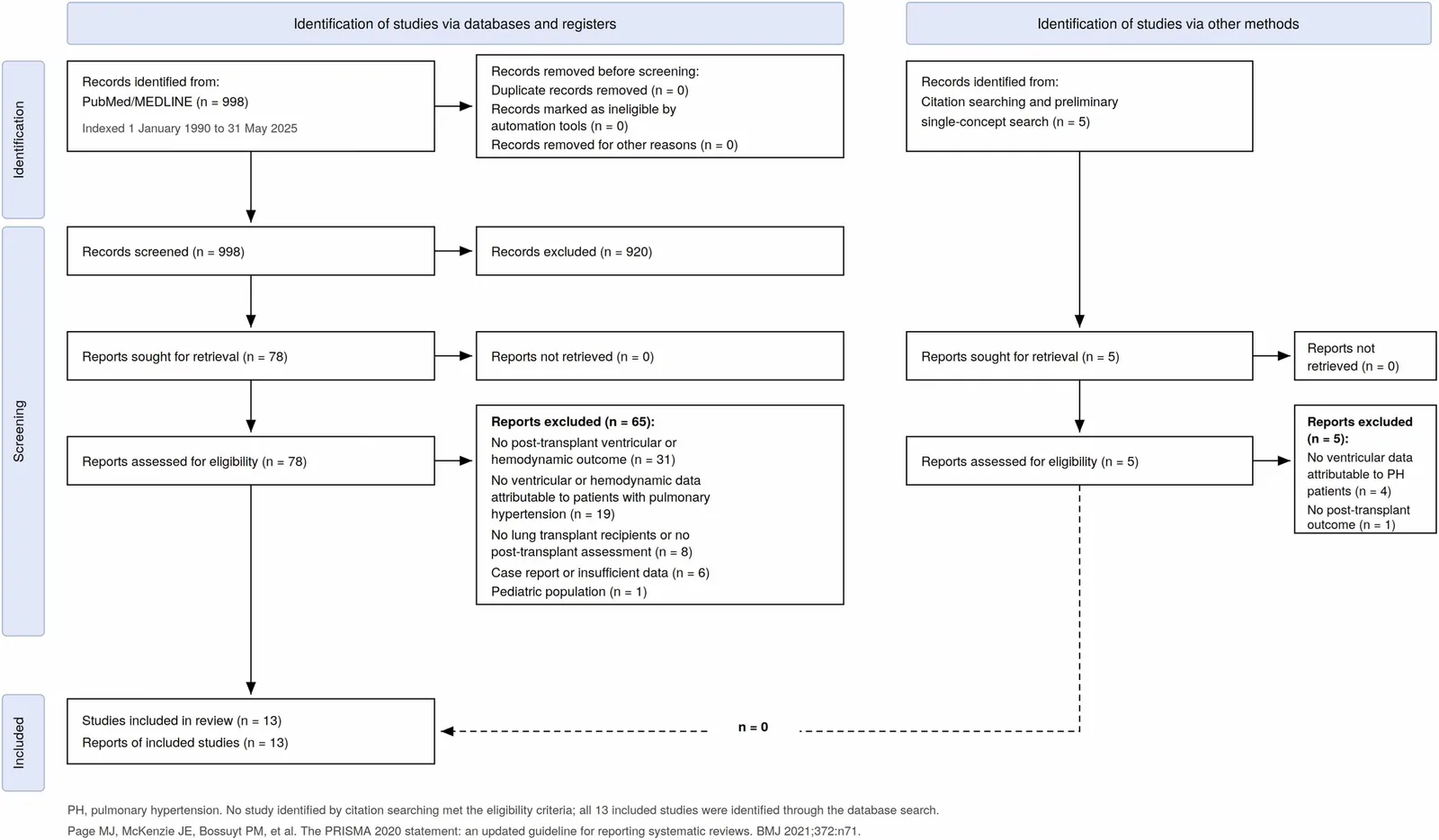
PRISMA 2020 flow diagram of study identification, screening and inclusion. Records were identified from PubMed/MEDLINE, restricted to those indexed between 1 January 1990 and 31 May 2025. Five further reports were identified through citation searching; none met the eligibility criteria. PH, pulmonary hypertension.

Ten studies were retrospective and three prospective, and all were single-center. Sample sizes contributing paired ventricular measurements ranged from 10 to 205. Two reports draw on the same Hannover program and two on the same Okayama program, so the number of unique patients is smaller than the sum of the reported sample sizes and cannot be determined precisely from the published data. Two studies contribute indirect evidence: Habedank included 7 patients with PH among 20 recipients without reporting them separately, and Aggarwal defined PH as a mean pulmonary artery pressure above 20 mmHg in a cohort whose mean was 30.5 mmHg at a mean age of 56.6 years. Study characteristics, principal findings, and overall risk-of-bias ratings are given in Table 1.

**Table 1:** Characteristics and principal findings of the 13 included studies

**Study**	**Design; setting; risk of bias**	**Population; n**	**Procedure; support**	**Assessment; timing**	**Key RV findings**	**Key LV and other findings**	**ECMO / support findings**
Katz 1996^13^	Prospective observational; single center (Pittsburgh). Risk of bias: moderate	32 consecutive LTx recipients stratified by pre-transplant PASP: severe PH ≥75 mmHg (n = 10), intermediate 40–74 mmHg (n = 11), no PH <40 mmHg (n = 11). Mixed etiology (PPH, Eisenmenger, sarcoidosis, others)	Single or bilateral LTx	Transesophageal echocardiography and invasive hemodynamics, immediately before and after implantation (intraoperative only)	Severe PH group: PASP 115±26 → 45±19 mmHg; mean PAP 76±14 → 31±11 mmHg (both P < 0.05). RV end-diastolic area, end-systolic area and eccentricity index all decreased. RV fractional area change did NOT change consistently and did not differ between groups	Normalization of septal geometry. No LV functional data reported	Not reported
Kasimir 2004^5^	Retrospective observational case series; single center (Vienna). Risk of bias: moderate to high	17 consecutive patients with advanced primary pulmonary hypertension, 2000–2002; 10 females. Median age 30 y (16–53). All NYHA III–IV. 14 survivors assessed at 3 months	Isolated bilateral LTx in all; intraoperative VA-ECMO in all 17 (femoral n = 13, central n = 4)	Echocardiography (dimensions, TR, LVEF) and right heart catheterization (RVEF, CI, mean PAP); before and 3 months after LTx	RVEF 32.7±16.7 → 67.3±4.7%; RV area 32.34±3.27 → 18.02±1.71 cm²; RV wall thickness 1.13 → 1.01 cm; TV annulus 4.36±0.69 → 3.18±0.43 cm; RV basal 5.51 → 3.46 cm; TR grade 2.9±1.0 → 0.46±0.41; septum returned to physiological position in all (all P < 0.005)	LVEF 69.9±8.5 → 73.8±4.1% (P = 0.023); cardiac index 2.2±0.8 → 4.1±1.28 L/min/m² (P < 0.005); mean PAP 61.5±16.5 → 17.4±3.7 mmHg (P < 0.005)	All transplants performed on ECMO. Explanted within 24 h in 16 of 17; 3 days in 1. Stated protocol: ECMO retained for the first 12 h to reduce pulmonary flow and allow gradual cardiac adaptation. 3-month mortality 17.5% (3 deaths)
Toyooka 2009^14^	Retrospective comparative cohort; single center (Okayama). Risk of bias: moderate	33 living-donor lobar LTx recipients: PAH n = 12, non-PAH n = 21	Living-donor lobar LTx	Radionuclide ventriculography and right heart catheterization (NOT echocardiography); pre-LTx, 2 months, 6 months, then annually to 3 years	PASP 93.1±6.7 → 25.7±1.8 mmHg at 2 months (non-PAH 22.4). RVEF 29.8±7.0% pre (vs 49.7±3.3, P = 0.010) → 57.3±5.1% at 2 months (equal to non-PAH, P = 0.86) → 65.1±2.4% at 6 months (higher than non-PAH 57.2±2.0, P = 0.020); 64.0 / 66.2 / 66.6% at 1 / 2 / 3 y	LVEF 49.9±6.6% pre (vs 65.2±1.9, P = 0.068) → 50.9±3.7% at 2 months (vs 65.4±2.8, P = 0.0038) → 64.1±3.2% at 6 months (vs 63.0±2.4, P = 0.78); 61.4 / 66.0 / 70.9% at 1 / 2 / 3 y. CI 2.66±0.12 at 2 months (vs 3.13±0.15, P = 0.037). LV recovery lagged RV recovery by 4–10 months	Not reported
Habedank 2011^15^	Prospective observational case series; single center (Berlin). Risk of bias: high for this question (indirect population)	20 bilateral LTx recipients (9 women), age 46.0±13.0 y. Etiology: COPD n = 8, pulmonary hypertension n = 7, idiopathic fibrosis n = 3, other n = 2. Group 1 PAH status not stated	Bilateral LTx	Echocardiography, cardiopulmonary exercise testing and blood gases; pre-LTx and 3, 6, 12, 24 months	RVEDd 35.0 (IQR 22.5) → 31.0 (9.0) mm at 3 months; RVEF 43.3±18.7 → 56.7±7.5%; RV systolic pressure 53.6±28.3 → 26.2±5.2 mmHg (all P < 0.01). RVEDd correlated with VE/VCO₂ slope before but not after LTx	Not reported	Not reported
Gupta 2015^9^	Retrospective comparative cohort; single center (UT Southwestern). Risk of bias: high	16 patients with PAH transplanted July 2008 – July 2012; 11 of 16 WHO group 1 (the abstract states 10 — an internal inconsistency in the source). Compared LVD (n = 5) with non-LVD (n = 11)	LTx; cardiopulmonary bypass in 5 of 5 LVD patients and 7 of 11 non-LVD patients. ECMO not used	Transthoracic echocardiography, pre-transplant cardiac MRI (median 4 months before LTx), right and left heart catheterization; early postoperative period to 4 months	Pre-transplant cMRI RVEF 40.6±10.2% (non-LVD) vs 35.8±11.0% (LVD), P = 0.47; RVEDV 257.6±140.5 vs 238±92.9 mL, P = 0.95	LVD in 5 of 16 (31%), detected by TTE at a mean of 4.2 days (range 1–10). LVEF in the LVD group fell from 69.6% to 37.4±13.9% (P = 0.02) versus 61.3±12.8% in the non-LVD group. Pre-transplant cMRI LVEF 68.7±18.7 vs 53.2±15.2% (P = 0.08); LVEDV 106.5 vs 126.5 mL. All LVD resolved within 4 months	No ECMO. Cardiopulmonary bypass required in all 5 LVD patients
Tudorache 2015^16^	Retrospective cohort with two historical control groups; single center (Hannover). Risk of bias: moderate to high	23 consecutive BLTx for severe PH from March 2010 (BLTx-ECMO group). Historical controls: BLTx-ventilation n = 13 and HLTx. 4 of 23 bridged pre-transplant on awake VA-ECMO	BLTx on VA-ECMO, continued at least 5 days postoperatively; early extubation attempted under continued ECMO	Echocardiography, left atrial and Swan–Ganz catheters; pre-LTx, day 2, day 5, day of decannulation, 3 and 12 months	RVEF 34±10% → 42±10% (day 2) → 49±7% (day 5). Mean PAP 67±17 → 18±7 mmHg on day 2 (P < 0.0001), unchanged at day 5	LVEF 65±13% pre → 47±17% on day 2 (P = 0.02) → 60±10% on day 5 (P = 0.04). LVEDD 37±10 mm pre → 46±0 mm day 2 → 42±6 mm day 5 (P = 0.0008), stable thereafter. Cardiac output 4±1.6 → 5.6±0.7 L/min. Reducing ECMO flow on day 2 raised LA pressure and lowered systemic pressure	Median postoperative ECMO 8 days (range 5–19). Survival 100% at 3 months and 96% at 12 months. LA pressure response to flow reduction used as the weaning criterion
Gorter 2017^17^	Retrospective observational case series; single center (Groningen). Risk of bias: moderate	10 patients with group 1 PAH with paired pre- and post-LTx cardiac MRI, selected from 26 listed (16 excluded, mainly for missing imaging). IPAH 6, HPAH 1, drug-induced 1, PAH-CHD 1, PVOD 1. Median age 44.0 y	Isolated bilateral LTx; cardiopulmonary bypass in 8 of 10	Cardiac MRI (primary) and echocardiography; median 3.1 y (IQR 1.4–4.8) after LTx for MRI and 3.5 y (IQR 1.3–4.7) for echocardiography	RVEF 32±9 → 64±9%; RVEDVi 118±37 → 51±13 mL/m²; RV mass index 69±16 → 33±9 g/m²; SV/ESV 0.5 → 1.9; RV/LV volume ratio 2.3 → 0.8; RV/LV mass ratio 1.3±0.2 → 0.5±0.1; TV annulus area 9.8±2.7 → 4.6±1.6 cm²/m² (all P < 0.001). After LTx 1 patient had grade 1 TR and none had grade 2–3	LVEF 58±8 → 62±11% (P = 0.28); LVEDVi 53±19 → 61±11 mL/m² (P = 0.11); LV mass index 55±12 → 61±13 g/m² (P = 0.005); LV eccentricity index 2.8±1.0 → 1.1±0.1 (P < 0.001). Right atrial volume index DECREASED from 102 to 43 mL/m² (P = 0.007); left atrial volume index increased from 20 to 36 mL/m² (P = 0.08, not significant). 3 patients had an important late fall in LVEF, each with an identified or unexplained cause	Postoperative VA-ECMO in 3 of the 8 patients operated on bypass
Salman 2017^18^	Retrospective observational case series; single center (Hannover). Risk of bias: moderate	38 of 717 lung transplants (5%) performed for severe PH, May 2010 – January 2016	Bilateral LTx on groin-cannulated VA-ECMO in all; sternum-sparing thoracotomy in 36 (95%); awake ECMO extended at least 5 days	Echocardiography preoperatively, at discharge, 3 months and 12 months	Proximal RV outflow diameter 48 (43–51) → 25 (23–27) mm at 12 months (P < 0.05). Tricuspid regurgitation had disappeared in all patients at 1 year	LV end-diastolic dimension 40 (32–44) → 45 (44–47) mm at 12 months (P < 0.05)	Prophylactic perioperative VA-ECMO protocol with early extubation (median 2 days). Survival at 3 months, 1 year and 5 years not different from transplants for other indications (P = 0.45)
Sarashina 2017^19^	Retrospective observational case series; single center (Okayama). Risk of bias: moderate to high	12 consecutive patients with PAH on combination targeted therapy, drawn from 32 PAH patients among 136 lung transplants performed 1998–2014	LTx	Echocardiography and right heart catheterization; before LTx and at 3 and 12 months. Denominators vary by parameter (n = 5 to n = 12)	TRPG 80±17 → 18±10 mmHg at 3 months (P < 0.001, n = 10) and 20±12 at 12 months. RVFAC 15±6 → 34±9% at 3 months (P = 0.003, n = 6) → 52±13% at 12 months (P < 0.001, n = 5). RV basal diameter 50±13 → 30±6 mm. RV wall thickness 8.7±1.1 → 8.2±3.0 mm at 3 months (P = 0.799) → 6.1±1.7 mm at 12 months (P = 0.017). Pericardial effusion 11.5±11.1 → 0.5±1.5 mm (P = 0.001)	LVEF (Teichholz) 71±11 → 61±6% at 3 months (P = 0.014, n = 11), recovering to 69±5% at 12 months (P = 0.789). LV stroke work index 28.9±8.7 → 45.1±6.3 g/m²/beat (P < 0.001). RA major diameter 6±2 → 4±1 cm (P = 0.009)	Not reported
Verbelen 2018^6^	Retrospective comparative cohort; single center (Leuven). Risk of bias: moderate	24 bilateral LTx for PAH compared with 27 pulmonary endarterectomies for CTEPH (with PVR reduction ≥800 dyn·s·cm⁻⁵)	Bilateral LTx (PAH arm); pre-transplant atrial septostomy in a subset	Echocardiography before and approximately 3 weeks after surgery	RV diastolic area 37±10 → 18±5 cm² (P < 1×10⁻⁵), a larger fall than after endarterectomy (P < 0.001 between groups)	PAH arm: LV mass 104±39 → 159±53 g (P < 1×10⁻⁴); LVEDD 37±7 → 43±6 mm (P < 0.001); LV posterior wall 9±2 → 10±3 mm (P < 0.05); LVEF 71±11 → 65±11% (P < 0.05); cardiac index 3.08±0.84 → 2.75±0.93 (P = 0.146, and lower than the endarterectomy arm, P < 1×10⁻¹⁶). Two patients developed post-transplant LV failure; both were diagnosed with PH before age 12, had a diagnosis-to-surgery interval 2.5 times longer, smaller LV mass and higher pre-transplant PVR. Pre-transplant atrial septostomy increased LV dimensions, stroke volume and cardiac index	Not reported
Schuba 2019^20^	Retrospective comparative cohort; single center (Munich). Risk of bias: high for ventricular outcomes	343 lung transplants October 2010 – August 2016, of which 54 had severe PH (IPAH n = 13; PH secondary to lung disease n = 41). Echocardiography available in 49 before and 29 after transplantation	Sequential bilateral LTx with individualized extracorporeal circulation	Echocardiography, performed once during follow-up at non-standardized intervals	TAPSE 16.5±4.8 → 20.5±5.9 mm (P = 0.002); RV basal diameter 47.3±10.9 → 37.9±6.7 mm (P = 0.001); RV systolic pressure 62.8±27.4 → 26.1±10 mmHg (P = 0.001). TR grade 2–3 in 36 of 48 before versus 2 of 29 after	LVEF 67±11 → 64±7% (not significant). RA diameter 46.4±12.5 → 41.6±10 mm (not significant)	Individualized extracorporeal circulation; Veno-venous preferred over Veno-arterial configuration postoperatively in some patients
Otto 2022^21^	Retrospective comparative cohort; single center (Alfred, Melbourne). Risk of bias: low to moderate	50 patients transplanted for PAH, 2006–2019. Compared LVD (n = 21) with non-LVD (n = 29). No loss to follow-up	LTx; ECMO in 26 (52%), categorized as preoperative, prophylactic or rescue	Echocardiography or strict clinical criteria; postoperative and at 12 months	Not reported separately	Postoperative LVD in 21 of 50 (42%), defined as an LVEF fall ≥15% or a one-grade reduction in LV function. Twelve-month survival and LV function did not differ between LVD and non-LVD groups	ECMO in 52%; prophylactic VA-ECMO use increased across the study period. Twelve-month survival 48 of 50 (96%)
Aggarwal 2024^22^	Retrospective comparative cohort; single center (Minnesota). Risk of bias: low, but highly indirect population	205 recipients with pulmonary hypertension (mean PAP >20 mmHg) drawn from 476 primary lung transplants, January 2011 – September 2021. Mean PAP 30.5±9.8 mmHg; mean age 56.6±11.9 y. Predominantly not group 1 PAH	Single or bilateral LTx	Echocardiography before and within 1 year after transplantation; diastolic function graded by the ASE 2016 guideline	Not reported	Diastolic dysfunction present in 169 before transplantation and resolved in 148 after (P < 0.001). Pre-transplant diastolic dysfunction was not associated with 1-year mortality, major adverse cardiac events or BOS-free survival. LVEF <55% in 9 (4.3%) at baseline	Not reported

BOS, bronchiolitis obliterans syndrome; BLTx, bilateral lung transplantation; CI, cardiac index; cMRI, cardiac magnetic resonance imaging; COPD, chronic obstructive pulmonary disease; CTEPH, chronic thromboembolic pulmonary hypertension; ECMO, extracorporeal membrane oxygenation; HLTx, heart–lung transplantation; HPAH, heritable pulmonary arterial hypertension; IPAH, idiopathic pulmonary arterial hypertension; LA, left atrial; LTx, lung transplantation; LV, left ventricular; LVD, left ventricular dysfunction; LVEDD, left ventricular end-diastolic diameter; LVEDVi, left ventricular end-diastolic volume index; LVEF, left ventricular ejection fraction; PAH, pulmonary arterial hypertension; PAP, pulmonary artery pressure; PASP, pulmonary artery systolic pressure; PH, pulmonary hypertension; PPH, primary pulmonary hypertension; PVOD, pulmonary veno-occlusive disease; PVR, pulmonary vascular resistance; RA, right atrial; RV, right ventricular; RVEDd, right ventricular end-diastolic diameter; RVEDVi, right ventricular end-diastolic volume index; RVEF, right ventricular ejection fraction; RVFAC, right ventricular fractional area change; SV/ESV, stroke volume to end-systolic volume ratio; TAPSE, tricuspid annular plane systolic excursion; TR, tricuspid regurgitation; TRPG, trans tricuspid regurgitation pressure gradient; TTE, transthoracic echocardiography; TV, tricuspid valve; VA, Veno-arterial.

### Risk of bias and synthesis

Two reviewers (A.S.M & P.G) independently extracted study characteristics, population details, imaging modality and timing, and all reported right and left ventricular, atrial and hemodynamic outcomes into a piloted form, with discrepancies resolved by discussion; extracted values were checked against the full text of each primary publication. Methodological quality and risk of bias were evaluated independently by two assessors utilizing the standardized Joanna Briggs Institute critical appraisal tools, with discrepancies settled via mutual consensus. The risk of bias was moderate or high in 11 of the 13 included studies (Table 1; Supplementary Tables S3 and S4). Four problems recur. Attrition affects several of the largest effect estimates: Kasimir restricted its paired analysis to the 14 patients surviving to three months, Schuba imaged 49 patients before transplantation and 29 afterwards, and Gorter included 10 of 26 eligible patients, selected largely by whether paired imaging existed. Denominators also vary within studies, falling to five patients for the largest reported improvement in right ventricular fractional area change. Control group design is a limitation where comparisons exist: Tudorache used historical controls transplanted earlier at the same center, and Verbelen contrasted two different diseases treated with two different operations. Finally, the timing of assessment ranged from the intraoperative period alone to a median of 3.1 years, which limits comparison across studies more than any single methodological weakness. Otto and Aggarwal were the most rigorously conducted studies, although Aggarwal’s population limits its applicability to PAH.

## RV recovery after lung transplantation

In PAH, sustained elevation of pulmonary vascular resistance imposes an afterload the right ventricle was not built to carry, producing dilation, hypertrophy and a fall in RV ejection fraction (RVEF), and impairing LV geometry through ventricular interdependence.^12^, ^19^ Progression to RV–PA uncoupling despite medical therapy is a key determinant of transplant eligibility. Invasive pressure–volume loop analysis remains the gold standard for assessing RV–PA coupling, but noninvasive measures such as the TAPSE to PASP ratio have emerged as prognostic markers, and how these normalize after LTx remains an active area of investigation.^23^

Reverse remodeling of the RV after transplantation was the most consistent finding in this review. Every included study that measured RV structure or function reported improvement in the same direction, irrespective of imaging modality, transplant technique or era (Table 1). The process begins within the operation itself. Katz et al. documented an immediate reduction in RV area and in the eccentricity index once the donor lungs were perfused, although contractile function, measured as fractional area change, did not improve consistently at that stage; it was persistent depression of contractility, rather than persistent dilation, that marked the patients who died early.^13^ The magnitude of subsequent recovery is large and concordant across methods. Whether measured by radionuclide ventriculography at two months,^14^ by catheterization at three months,^5^ or by CMR a median of three years after transplantation,^17^ RVEF approximately doubled from severely depressed pre-transplant values into the normal range, and CMR demonstrated complete normalization of RV volume, mass and trabeculation irrespective of when postoperative imaging was performed.^17^ Echocardiographic series describe the same direction of change, with concurrent normalization of RV systolic pressure.^15^, ^20^ Given the prognostic weight carried by RVEF and the position of CMR as the reference standard for ventricular volumes and mass,^24^, ^25^ these findings support a role for CMR in post-transplant surveillance.^26^

The time course of recovery is better defined than is generally appreciated. Serial measurements in the first postoperative week show RVEF rising within days of implantation^16^; by two to three months it had reached the normal range in every series that measured it, with no further change at one, two and three years.^14^ TAPSE and RV end-diastolic dimensions improved in parallel.^20^ Not all components recover together, however. Chamber size and systolic function normalize within the first three months, but RV wall thickness is unchanged at that point and regresses only by twelve months,^19^ and residual hypertrophy was still detectable in patients whose chamber size and function had already normalized.^5^ Hypertrophy regresses more slowly than dilation.

Tricuspid regurgitation resolved in parallel with chamber size, from severe before transplantation to trivial or absent afterwards, with no more than mild residual regurgitation at long-term follow-up.^5^, ^17^, ^20^ Because annular size tracked RV end-diastolic volume,^17^ functional regurgitation from annular dilation rather than intrinsic valve disease is the most likely mechanism, and the valve can be expected to recover with the ventricle.

Clinical factors including age, PAH subtype, comorbidities and the Registry to Evaluate Early and Long-Term PAH Disease Management (REVEAL) risk score influence recovery, and earlier age at diagnosis is associated with worse outcomes through more prolonged exposure to elevated afterload.^6^ Use of prophylactic VA-ECMO varies between patients and between centers.^21^ Nevertheless, most studies confirm that reducing PVR with LTx restores right ventricular function in the majority of recipients.^20^

## Left ventricular adaptation after lung transplantation

The rapid reduction in PVR after LTx represents more than isolated RV recovery; it initiates a cascade of hemodynamic changes affecting both ventricles. In severe PAH, chronic RV pressure overload and ventricular interdependence contribute to LV compression, reduced LV filling and adverse LV remodeling. Following transplantation, abrupt normalization of PVR reduces afterload and permits recovery of RV output and geometry. The resulting increase in transpulmonary flow and venous return, together with relief of septal displacement and pericardial constraint, increases LV preload and exposes a chronically underfilled and remodeled LV to substantially different loading conditions. The early postoperative period therefore represents a transition from RV pressure overload and LV underfilling to increased biventricular filling and output, and provides the physiologic framework for the transient LVD observed in some patients after LTx^24^, ^26^, ^27^, ^28^ (Figure 2).

**Figure 2:**
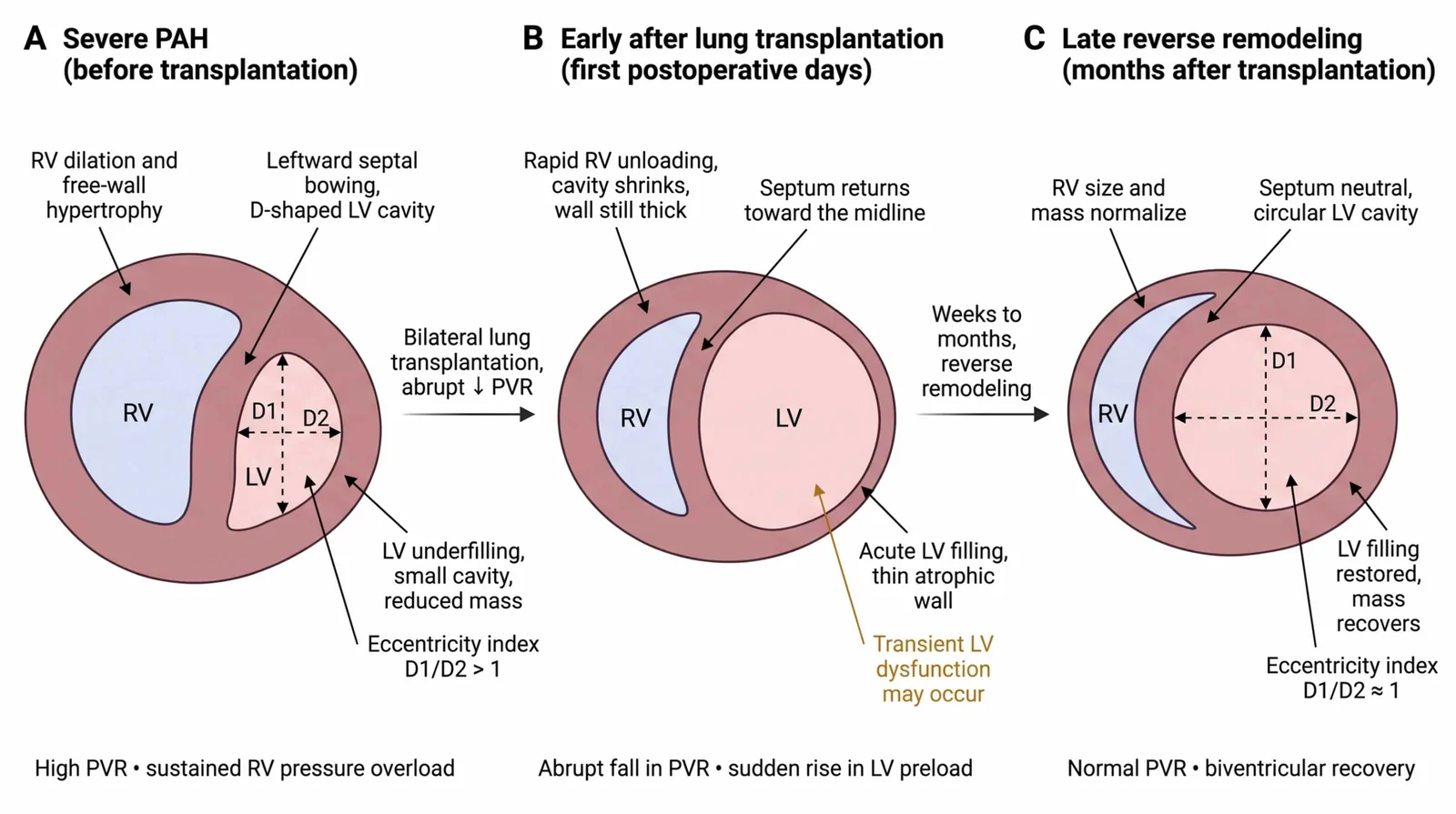
Ventricular geometry in severe pulmonary arterial hypertension before and after lung transplantation, shown in a short-axis cross-section at the mid-ventricular level. (A) Severe PAH. Sustained pressure overload dilates the RV and thickens its free wall; the interventricular septum is displaced leftward and bows into the LV, which becomes D-shaped, small and underfilled, with reduced mass. The eccentricity index (D1/D2, the ratio of the LV diameter parallel to the septum to the diameter perpendicular to it) is greater than 1. (B) Early after lung transplantation. The abrupt fall in pulmonary vascular resistance unloads the RV within days: the RV cavity shrinks although its wall remains thick, the septum returns toward the midline, and the LV cavity enlarges acutely as preload is restored to a ventricle whose wall is still thin and atrophic; transient LV dysfunction may occur during this phase. (C) Late reverse remodeling. RV size and free-wall thickness normalize, the septum is neutral, the LV is circular with restored filling and mass, and the eccentricity index approaches 1. Conceptual schematic; not to scale. LV, left ventricle; PAH, pulmonary arterial hypertension; PVR, pulmonary vascular resistance; RV, right ventricle. Created in BioRender. Mohammed, A. (2026) https://BioRender.com/6whg895.

LVD is a recognized but less well understood complication of LTx for PAH.^9^ The abrupt normalization of PVR increases LV preload, potentially overwhelming an underfilled and remodeled ventricle,^6^, ^12^, ^29^ and cessation of mechanical ventilation may further augment venous return, contributing to transient pulmonary edema and increased vasopressor requirements100053^9^ Every study that imaged the LV within the first postoperative days or weeks recorded a fall in systolic function. Serial echocardiography showed LVEF reaching its nadir around the second postoperative day, accompanied by acute enlargement of the LV cavity as the ventricle filled, with substantial recovery by day five.^16^ Clinically evident LVD, with hemodynamic compromise requiring escalation of support, affected roughly one third to two fifths of recipients depending on the definition applied; it typically declared itself within the first postoperative week and resolved within months.^9^, ^21^ A milder reduction in LVEF was still detectable at three weeks^6^ and at three months,^19^ by which time it had recovered in most patients.

Studies assessing later time points found no residual deficit. By three months, LVEF and cardiac index had risen above their pre-transplant values^5^; at a median of three years, LVEF was unchanged from baseline on CMR^17^; and recipients who had developed early LVD could not be distinguished from those who had not at twelve months.^21^ Toyooka et al. captured the whole trajectory within a single cohort: LVEF remained lower than in non-PAH recipients at two months despite complete RV recovery, normalized by six months, and continued to rise over three years.^14^ Pre-transplant diastolic dysfunction resolved in the great majority of recipients within the first year.^22^

The apparent inconsistency in the LV literature is largely explicable, and reflects when the ventricle was imaged and how dysfunction was defined rather than genuine disagreement between studies. Every series imaging within the first postoperative week found a fall in ejection fraction; every series imaging at three months or later found recovery; and Toyooka et al. captured both within a single cohort, showing that LV recovery lags RV recovery by four to ten months.^14^ Reported incidence differs for the same reason: Gupta et al. required acute cardiopulmonary compromise together with a fall in systolic function and identified LVD in about one third of recipients, whereas Otto et al. accepted either a 15% fall in LVEF or a one-grade qualitative decline and identified LVD in more than two fifths.^9^, ^21^ The two figures describe the same phenomenon at different thresholds. Recovery, where it was followed for long enough, was close to universal. Taken together, these findings suggest that early LVD after transplantation for PAH is not an isolated complication but the predictable response of a chronically underfilled ventricle to the sudden restoration of normal venous return.

Structural findings support underfilling as the mechanism. LV mass, the variable most directly reflecting chronic underloading, increased after transplantation in both studies that measured it,^6^, ^17^ while the LV eccentricity index normalized, confirming restoration of septal geometry.^17^ Manders et al. found approximately 30% cardiomyocyte atrophy in PAH and postulated that contractile dysfunction of atrophic cardiomyocytes re-exposed to normal filling pressures contributes to early postoperative problems.^12^ Consistent with that account, the two recipients who developed frank LV failure had been diagnosed before the age of 12, had a diagnosis-to-surgery interval 2.5 times longer than the rest of the cohort, and had smaller LV mass and higher pre-transplant PVR^6^: underfilled for longer, unloaded more abruptly. Beta blockers and angiotensin-converting enzyme inhibitors have been used to support LV function in the immediate perioperative period.^9^ Evidence on LV diameter and mass nevertheless remains inconsistent,^20^, ^30^ and predictors of maladaptive response have not been established.

## Atrial Changes Post-Lung Transplantation

Data on atrial remodeling after LTx in PAH are limited, and the two atria behave differently. Gorter et al. found that right atrial volume index fell from 102 to 43 mL/m² after transplantation (P = 0.007), while left atrial volume index rose from 20 to 36 mL/m², a change that did not reach significance (P = 0.08).^17^ Sarashina et al. similarly reported a reduction in right atrial major diameter from 6 to 4 cm (P = 0.009),^19^ and Schuba et al. observed a smaller, non-significant fall in right atrial diameter.^20^ This divergence is consistent with the underlying physiology: right atrial dilation reflects pressure overload and regresses once that load is removed, whereas left atrial volume rises as LV filling is restored. Whether left atrial enlargement after transplantation represents physiological adaptation or residual diastolic impairment has not been established.

## Modulating the Hemodynamic Transition with VA-ECMO

The role of ECMO after LTx for severe PAH should be understood in the context of the abrupt change in ventricular loading conditions that occurs after transplantation.^31^ By reducing RV preload and supporting systemic perfusion and oxygen delivery, VA-ECMO can provide a physiologic bridge while RV function recovers and LV filling increases. However, retrograde arterial flow can increase LV afterload and may contribute to LV distension when LV function is impaired; therefore, circuit flow and the need for LV unloading must be individualized. These competing effects are important because VA-ECMO can partially uncouple the normal series relationship between RV output, pulmonary blood flow, and LV preload, potentially obscuring the degree of native RV recovery until support is reduced.^32^ (Figure 3).

**Figure 3:**
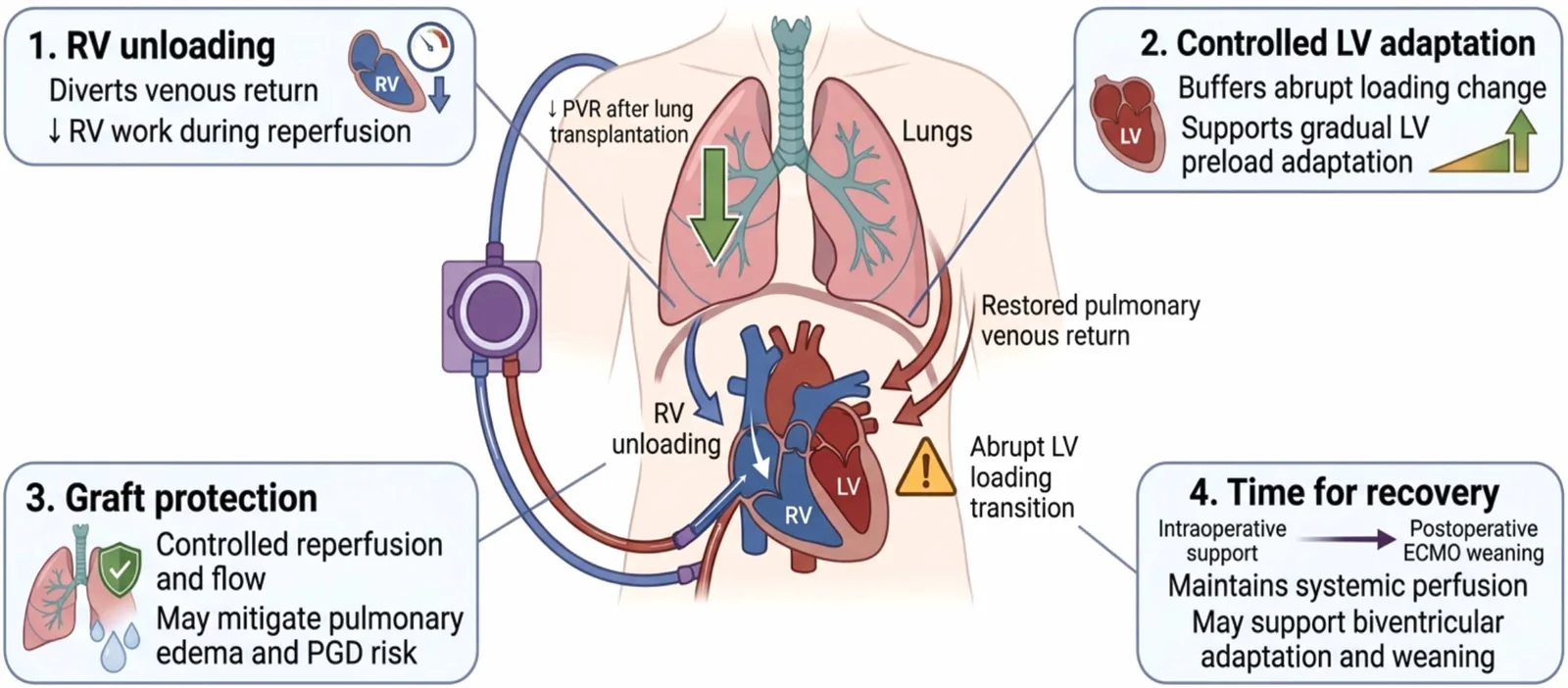
Role of perioperative venoarterial extracorporeal membrane oxygenation (VA-ECMO) during the hemodynamic transition after lung transplantation for severe pulmonary arterial hypertension. Implantation of the donor lungs abruptly lowers pulmonary vascular resistance and restores pulmonary venous return, exposing the left ventricle to a sudden increase in loading. VA-ECMO modifies this transition in four ways: **(1)** it diverts a portion of venous return and reduces right ventricular work during reperfusion; **(2)** it buffers the abrupt change in loading conditions and supports gradual adaptation of left ventricular preload; **(3)** it allows controlled reperfusion and graft flow and may mitigate pulmonary edema and primary graft dysfunction; and **(4)** it maintains systemic perfusion from the intraoperative period through postoperative weaning and may support biventricular adaptation. Conceptual schematic; no controlled study has tested these effects. ECMO, extracorporeal membrane oxygenation; LV, left ventricle; PGD, primary graft dysfunction; PVR, pulmonary vascular resistance; RV, right ventricle. Created in BioRender. Mohammed, A. (2026) https://BioRender.com/5gsrdd3.

Recent ISHLT consensus statements provide detailed recommendations for ECLS across the LTx continuum, including preoperative bridging, intraoperative implementation, and postoperative management; the postoperative statement specifically addresses extended support after transplantation for Group 1 PAH.^33^, ^34^, ^35^ General management is therefore not restated here. Instead, the evidence relating extracorporeal support to ventricular outcomes in PAH is considered separately for each phase in which it is used, because the physiologic objective of support differs between them.

### Preoperative support as a bridge to transplantation

In candidates with PAH who deteriorate on the waiting list, ECMO is used as a bridge to transplantation to unload the failing RV and to maintain systemic perfusion and oxygenation.^29^, ^33^ Cannulation under local anesthesia in awake, spontaneously breathing patients avoids the adverse effects of endotracheal intubation and positive-pressure mechanical ventilation on a pressure-overloaded RV, limits the infectious complications of prolonged sedation, and allows physical conditioning to continue before surgery.^29^, ^33^ Among patients bridged on ECMO, Kukreja et al. identified pre-cannulation RV failure, multiorgan dysfunction, high transfusion requirement, short stature and blood group B as factors associated with poorer outcomes, which argues for cannulation before secondary organ injury is established.^36^ None of the included studies reported ventricular outcomes separately for bridged patients, so the contribution of preoperative support to subsequent ventricular recovery cannot be separated from that of transplantation itself

### Intraoperative support

Intraoperative VA-ECMO is now preferred over cardiopulmonary bypass at many centers for transplantation in severe PAH.^34^ In the included studies, all recipients in the Vienna^5^ and Hannover^16^, ^18^ series were transplanted on VA-ECMO. Beyond hemodynamic stability during the operation, intraoperative support allows controlled reperfusion of the graft: by diverting part of the cardiac output through the circuit, it limits the pulmonary blood flow that the newly implanted lungs receive, which may mitigate reperfusion injury and early PGD.^18^, ^31^, ^34^ Because the fall in PVR occurs at the moment of implantation, intraoperative support also coincides with the instant at which LV preload rises, and this is the rationale for continuing it into the postoperative period

### Postoperative extension of support

Kasimir et al., from the Vienna program, retained ECMO for the first hours after transplantation specifically to limit pulmonary blood flow and allow gradual cardiac adaptation, and the same recipients showed normalization of chamber dimensions and ejection fraction at three months.^5^

The Hannover program developed this approach into a protocol of awake VA-ECMO continued for at least five days after bilateral LTx. Tudorache et al. attributed early PGD in part to the LV, proposing that diastolic failure and elevated filling pressures in long-standing PAH leave the ventricle unable to accommodate the increased cardiac output that follows implantation; in their series, reducing circuit flow raised left atrial pressure, which was used as the weaning criterion, and 90-day survival was 100%.^16^ In the larger Hannover series of Salman et al., extended postoperative VA-ECMO was accompanied by resolution of tricuspid regurgitation and normalization of RV outflow dimensions at one year, while LV end-diastolic dimension increased over the same interval, the change expected of a chronically underfilled ventricle permitted to fill gradually; mid-term survival did not differ from that of transplantation for other indications.^18^ These series have been read as support for perioperative VA-ECMO in place of combined HLT for severe PAH.^16^, ^18^ None, however, included a concurrent control group, and the decision to prolong support was made in every case by the teams reporting the outcomes, so the association between extended support and favorable ventricular recovery cannot be separated from selection by center protocol.

## Limitations

All 13 included studies are observational and single-center, and the largest series contributing paired ventricular measurements includes 50 patients. Eleven were judged at moderate or high risk of bias, most often because of incomplete or selective follow-up. Two studies contribute indirect evidence. Habedank included 7 patients with PH among 20 recipients without reporting them separately, and Aggarwal studied a cohort defined by a mean pulmonary artery pressure above 20 mmHg whose mean was 30.5 mmHg at a mean age of 56.6 years, a population that is predominantly not group 1. Both were retained because excluding cohorts of mixed etiology would have removed the only substantial data on diastolic function and on serial RV change beyond one year, but their findings should not be read as specific to PAH. Two further pairs of reports draw on overlapping single-center cohorts, so findings from those pairs are not independent. No study reported a randomized or concurrently controlled comparison of an extracorporeal support strategy. Heterogeneity in imaging modality, in the timing of post-transplant assessment, and in the definition of LVD precluded meta-analysis and limits comparison between studies.

## Conclusion

LTx for PAH initiates a rapid transition from chronic RV pressure overload and LV underfilling to restored pulmonary vascular conditions and progressive biventricular recovery. RV reverse remodeling is early, large and consistent across every included study. LV adaptation is slower and more variable, and early dysfunction is best understood not as an isolated complication but as the response of a chronically underfilled ventricle to the sudden restoration of normal venous return. VA-ECMO offers a means of moderating the rate of that transition, although no controlled study has yet tested whether doing so improves outcomes. Future work should define predictors of maladaptive LV response and identify which recipients benefit from prolonged perioperative support.

## Ethics approval statement

This review analyzed data from previously published studies and did not involve human participants, human tissue, or identifiable patient data. Institutional review board approval and informed consent were therefore not required. The review was conducted in accordance with the ethical principles of the 1964 Declaration of Helsinki and its later amendments.

## Funding

This research did not receive any specific grant from funding agencies in the public, commercial, or not-for-profit sectors.

## CRediT authorship contribution statement

Adil Sarvar Mohammed (A.S.M): Conceptualization, Resources, Investigation, Visualization, Writing—original draft, and Writing—review and editing, Priyatham Gurram (P.G): Conceptualization, Resources, Investigation, Writing—original draft, and Writing—review and editing, Sai Priyanka Mellacheruvu (S.P.M): Writing—original draft, Sai Prasanna Lekkala (SP.L): Writing—original draft, Maya Asami Takagi (M.A.T): Writing—original draft, Writing—review and editing, and Visualization, Sai Gautham Kanagala (S.G.K): Writing—original draft, Umera Yasmeen (U.Y): Writing—original draft, Hamdi Lababidi (H.L): Writing – review and editing, Zainab J Gandhi (Z.J.G): Writing—review and editing, Bilal Khan Mohammed (B.K.M): Writing—review and editing, Validation and Supervision, Shweta Kambali (S.K): Writing—review and editing, Rupak Desai (R.D): Conceptualization, Validation, Methodology, Writing—review and editing, Shrinivas Kambali (S.K.2): Investigation and Writing—editing, Validation and Supervision

## Data availability statement

The search strategy, eligibility criteria, and risk-of-bias appraisals supporting this review are provided in the Supplementary Material. All data analyzed were extracted from previously published studies, which are cited in the reference list. No new patient-level data were generated.

## Declaration of Generative AI and AI-assisted technologies in the writing process

Generative artificial intelligence was used solely to assist with language editing and improve the clarity of the manuscript. All content was critically reviewed and verified by the authors, who take full responsibility for the accuracy and integrity of the work.

## Declaration of Competing Interest

The authors declare that they have no known competing financial interests or personal relationships that could have appeared to influence the work reported in this paper.

## Footnotes

Appendix A Supplemental data associated with this article can be found in the online version at doi:10.1016/j.jhlto.2026.100715.
