# Supplementary material for: Effect of lung transplantation on biventricular function in pulmonary arterial hypertension: A systematic review
Source: JHLT Open. 2026 Oct 27;15:100715. doi: 10.1016/j.jhlto.2026.100715 (PMC13756534; doi:10.1016/j.jhlto.2026.100715)
Supplement: Supplementary file 1 [file mmc1.docx]

# Supplementary Material

**Contents**

- Supplementary Table S1. Search strategy
- Supplementary Table S2. Eligibility criteria (PICOS)
- Supplementary Table S3. Critical appraisal of comparative cohort studies
- Supplementary Table S4. Critical appraisal of before-and-after case series

# Supplementary Table S1. Search strategy

PubMed/MEDLINE was searched and restricted to records indexed between **1 January 1990 and 31 May 2025**. Medical Subject Headings and title/abstract terms for three concepts — pulmonary hypertension, lung transplantation, and ventricular structure, function or hemodynamics (the last including cardiac imaging and extracorporeal membrane oxygenation) — were combined with the Boolean operator AND. Truncation is indicated by an asterisk.

No language or publication-type filter was applied at the search stage; language and study-design restrictions were applied at screening. No duplicate records were removed, no automation tool was used, and no record was excluded before screening.

**Records returned by the combined search: 998.** A further 5 reports were identified through citation searching and a preliminary single-concept search. Screening and eligibility outcomes are shown in the PRISMA 2020 flow diagram (Figure 1).

## Line 1 — Pulmonary hypertension

"Hypertension, Pulmonary"[Mesh] OR "pulmonary arterial hypertension"[tiab]
OR "pulmonary hypertension"[tiab] OR PAH[tiab] OR IPAH[tiab]

## Line 2 — Lung transplantation

"Lung Transplantation"[Mesh] OR "Heart-Lung Transplantation"[Mesh]
OR "lung transplant"[tiab] OR "lung transplants"[tiab]
OR "lung transplantation"[tiab] OR "lung transplanted"[tiab]
OR "pulmonary transplantation"[tiab] OR "lung allograft"[tiab]
OR "lung allografts"[tiab]

## Line 3 — Ventricular structure, function, hemodynamics, imaging and extracorporeal support

"Ventricular Function"[Mesh] OR "Ventricular Dysfunction"[Mesh]
OR "Ventricular Remodeling"[Mesh] OR "Heart Ventricles"[Mesh]
OR "Stroke Volume"[Mesh] OR "Hemodynamics"[Mesh:NoExp]
OR "Echocardiography"[Mesh] OR "Magnetic Resonance Imaging, Cine"[Mesh]
OR "Extracorporeal Membrane Oxygenation"[Mesh]
OR "ventricular function"[tiab] OR "ventricular dysfunction"[tiab]
OR "ventricular failure"[tiab] OR "ventricular remodeling"[tiab]
OR "ventricular remodelling"[tiab] OR "ventricular recovery"[tiab]
OR "ventricular mass"[tiab] OR "ventricular volume"[tiab]
OR "ventricular volumes"[tiab] OR "ejection fraction"[tiab]
OR "right ventricle"[tiab] OR "left ventricle"[tiab]
OR "right ventricular"[tiab] OR "left ventricular"[tiab]
OR biventricular[tiab] OR "cardiac index"[tiab] OR "cardiac output"[tiab]
OR "tricuspid annular plane systolic excursion"[tiab] OR TAPSE[tiab]
OR "tricuspid regurgitation"[tiab] OR echocardiograph*[tiab]
OR "cardiac magnetic resonance"[tiab]
OR "cardiovascular magnetic resonance"[tiab] OR "cardiac MRI"[tiab]
OR "extracorporeal membrane oxygenation"[tiab] OR ECMO[tiab]
OR "extracorporeal life support"[tiab]

## Line 4 — Combined search, with date limits applied

(Line 1) AND (Line 2) AND (Line 3)
AND ("1990/01/01"[Date - Publication] : "2025/05/31"[Date - Publication])

**Records: 998**

## Supplementary searching

Reference lists of all included studies and of relevant consensus documents were hand-searched, and forward citation tracking was performed. Five additional reports were identified by these methods; none met the eligibility criteria.

# Supplementary Table S2. Eligibility criteria (PICOS)

| Element | Included | Excluded |
| --- | --- | --- |
| **Population** | Adults undergoing lung transplantation for pulmonary arterial hypertension, including primary or idiopathic pulmonary hypertension and Eisenmenger syndrome. Cohorts of mixed pulmonary hypertension etiology were eligible where patients with pulmonary hypertension formed a defined part of the transplanted cohort and ventricular outcomes were reported for that cohort, whether or not a pulmonary arterial hypertension subgroup could be separated | Pediatric cohorts; populations in which pulmonary hypertension was not identifiable; transplant candidates, waitlist or bridging cohorts without post-transplant assessment; isolated heart–lung transplantation |
| **Intervention** | Single, bilateral or living-donor lobar lung transplantation, with or without perioperative extracorporeal membrane oxygenation | — |
| **Comparator** | Pre-transplant baseline (within-patient), non-PAH transplant recipients, or alternative perioperative support strategies. A comparator was not required | — |
| **Outcomes** | *Primary:* post-transplant change in right ventricular structure and function (ejection fraction, dimensions, mass, fractional area change, tricuspid annular plane systolic excursion, pulmonary artery pressure, tricuspid regurgitation, RV–pulmonary artery coupling) and in left ventricular structure and function (ejection fraction, dimensions, mass, diastolic function, cardiac index, postoperative left ventricular dysfunction). *Secondary:* timing of ventricular recovery, septal and geometric change, and the association of perioperative venoarterial ECMO with ventricular adaptation, hemodynamic stability, primary graft dysfunction and early survival | Studies reporting only survival, graft or pre-transplant predictor data |
| **Study design** | Prospective or retrospective cohort studies, case–control studies, and case series of two or more patients | Single case reports; animal and experimental studies; reviews, guidelines, consensus statements, editorials, protocols, letters, and abstracts without extractable data |
| **Language** | English | Publications not available in English |
| **Dates** | 1 January 1990 to 31 May 2025 | — |

Reviews, guidelines and consensus statements were not eligible for inclusion but were used to inform context and for citation searching.

# Supplementary Table S3. Critical appraisal of comparative cohort studies

Joanna Briggs Institute Critical Appraisal Checklist for Cohort Studies. Y, yes; N, no; U, unclear; dash, not applicable.

**Items:** 1, groups similar and recruited from the same population; 2, exposures measured similarly across groups; 3, exposure measured in a valid and reliable way; 4, confounding factors identified; 5, strategies to deal with confounding stated; 6, participants free of the outcome at the start; 7, outcomes measured in a valid and reliable way; 8, follow-up reported and sufficient; 9, follow-up complete or incompleteness explained; 10, strategies to address incomplete follow-up used; 11, statistical analysis appropriate.

| Study | 1 | 2 | 3 | 4 | 5 | 6 | 7 | 8 | 9 | 10 | 11 | Overall |
| --- | --- | --- | --- | --- | --- | --- | --- | --- | --- | --- | --- | --- |
| Katz 1996 | Y | Y | Y | U | N | Y | Y | N | Y | — | Y | Moderate |
| Toyooka 2009 | Y | Y | Y | U | N | Y | Y | Y | U | N | Y | Moderate |
| Gupta 2015 | Y | Y | Y | U | N | Y | Y | Y | Y | — | U | High |
| Tudorache 2015 | N | Y | Y | Y | U | Y | Y | Y | Y | U | Y | Moderate to high |
| Verbelen 2018 | N | Y | Y | Y | U | Y | Y | N | U | U | Y | Moderate |
| Schuba 2019 | Y | Y | Y | U | N | Y | U | U | N | N | Y | High |
| Otto 2022 | Y | Y | Y | Y | U | Y | U | Y | Y | Y | Y | Low to moderate |
| Aggarwal 2024 | Y | Y | Y | Y | Y | Y | Y | Y | Y | Y | Y | Low |

Aggarwal was rated at low risk of bias on every item but contributes indirect evidence, because pulmonary hypertension was defined as a mean pulmonary artery pressure above 20 mmHg in a cohort whose mean was 30.5 ± 9.8 mmHg at a mean age of 56.6 years.

# Supplementary Table S4. Critical appraisal of before-and-after case series

Joanna Briggs Institute Critical Appraisal Checklist for Case Series. Y, yes; N, no; U, unclear.

**Items:** 1, clear criteria for inclusion; 2, condition measured in a standard and reliable way; 3, valid methods used to identify the condition; 4, consecutive inclusion; 5, complete inclusion; 6, demographics clearly reported; 7, clinical information clearly reported; 8, outcomes or follow-up results clearly reported; 9, presenting site or clinic demographics reported; 10, statistical analysis appropriate.

| Study | 1 | 2 | 3 | 4 | 5 | 6 | 7 | 8 | 9 | 10 | Overall |
| --- | --- | --- | --- | --- | --- | --- | --- | --- | --- | --- | --- |
| Kasimir 2004 | Y | Y | Y | Y | Y | Y | Y | N | Y | U | Moderate to high |
| Habedank 2011 | N | Y | Y | Y | Y | Y | U | Y | Y | Y | High for this question |
| Gorter 2017 | Y | Y | Y | Y | N | Y | Y | Y | Y | Y | Moderate |
| Salman 2017 | Y | Y | Y | Y | N | Y | Y | Y | Y | U | Moderate |
| Sarashina 2017 | Y | Y | Y | Y | Y | Y | Y | U | Y | U | Moderate to high |

Habedank was rated as high risk for the question addressed by this review because only 7 of 20 transplant recipients had pulmonary hypertension and no subgroup analysis was reported; the study is otherwise prospective, consecutive and complete.
